# Target Gene-Based Association Study of High Mobility Group Box Protein 1 in Intracranial Aneurysms in Koreans

**DOI:** 10.3390/brainsci14100969

**Published:** 2024-09-26

**Authors:** Eun Pyo Hong, Sung Woo Han, Bong Jun Kim, Dong Hyuk Youn, Jong Kook Rhim, Jin Pyeong Jeon, Jeong Jin Park

**Affiliations:** 1 Institute of New Frontier Research, Hallym University College of Medicine, Chuncheon 24254, Republic of Korea; ephong0305@hallym.ac.kr (E.P.H.); hsw4070@naver.com (S.W.H.); luckykbj@naver.com (B.J.K.); zk61326@naver.com (D.H.Y.); 2 Department of Neurosurgery, Jeju National University College of Medicine, Jeju 63241, Republic of Korea; nsrhim@gmail.com; 3 Department of Neurosurgery, Hallym University College of Medicine, Chuncheon 24253, Republic of Korea; jjs6553@daum.net; 4 Department of Neurology, Konkuk University Medical Center, Seoul 05030, Republic of Korea

**Keywords:** expression of HMGB1 gene, HMGB1 protein level in plasma, intracranial aneurysm, variants in HMGB1 gene

## Abstract

**Objective:** We investigated the effect of high mobility group box 1 (HMGB1) on intracranial aneurysms (IAs) by analyzing single-nucleotide polymorphisms (SNPs) based on genome-wide association study (GWAS) data. HMGB1 mRNA and protein expression levels in plasma were also analyzed. **Methods**: This study was a comprehensive analysis of a GWAS dataset, including 250 patients with IAs and 294 controls. The HMGB1 gene region was targeted within SNP rs3742305 ± 10 kbp. Multivariate logistic regression analysis determined its association with IAs after adjusting for relevant clinical factors. HMGB1 mRNA expression was analyzed in the plasma of 24 patients selected from the GWAS dataset. The HMGB1 protein was analyzed by Western blotting. **Results:** A total of seven polymorphisms, including rs1360485, rs185382445, rs2039338, rs1045411, rs3742305, rs2249825, and rs189034241, were observed. Two SNPs, including rs1045411 (UTR-3) and rs3742305 (intron), showed strong linkage disequilibrium (r^2^ = 0.99). However, none of the seven SNPs associated with IAs had an adjusted *p*-value of < 0.0016 on multiple comparison analysis. HMGB1 mRNA levels (2^−ΔCt^) did not differ significantly between patients with IAs and the control subjects [1.07 (1.00–1.15) in patients with IAs vs. 1.05 (0.94–1.12) in controls; *p* = 0.67)]. Also, no significant difference in the degree of plasma HMGB1 protein expression was seen between the two groups (*p* = 0.82). **Conclusions:** The number of SNPs associated with HMGB1 and the degree of HMGB1 mRNA and protein expression were not significantly different between patients diagnosed with IAs and the controls.

## 1. Introduction

Intracranial aneurysms (IAs) are the leading cause of hemorrhagic stroke, accounting for 70–85% of non-traumatic subarachnoid hemorrhages (SAHs) [1]. The mortality rate of subarachnoid hemorrhage due to aneurysms is between 23 and 51% [1,2,3], and disability rates of 10–20% have been reported [4]. The overall prevalence of IAs with a saccular or berry-like appearance was reported to range from 0.2 to 5% in the general population [5,6,7]. Additionally, the number of diagnoses tends to increase with age and the greater availability of radiological tests [7,8]. Asaithambi et al. [7] reported that the highest incidence rates of unruptured intracranial aneurysms (UIAs) and ruptured IAs resulting in SAH were observed in those between the ages of 75 and 84 (61.6 per 100,000 persons) and over 85 years (30.1 per 100,000 persons), respectively, in the Minnesota population. UIAs can be asymptomatic while maintaining non-progressive morphological changes during follow-up, or they can lead to SAH as the shape of an IA changes or it grows in size. The IA rupture rate was estimated at 1.6% but increased depending on IA size, shape, and location [8,9]. Although magnetic resonance angiography (MRA) has been used as a regular check-up tool to identify the growth of or changes in IAs, many patients with IAs hesitate to undergo MRA in actual clinical practice due to cost. Therefore, if IA growth and rupture can be predicted by blood tests, frequent monitoring of IA changes can be conducted, and unnecessary MRA costs can be avoided.

The development and growth of IAs are closely associated with inflammation, resulting in endothelial dysfunction and abnormal extracellular matrix (ECM) remodeling in response to hemodynamic stress [10]. Histological investigations of IAs revealed arterial wall inflammation with pathological findings, such as ECM degradation, proteolysis, and apoptosis [10,11]. The expression of several pro-inflammatory cytokines, such as interleukin-6 (IL-6), tumor necrosis factor-α (TNF-α), and high mobility group box 1 (HMGB1), is increased in IA tissues compared to normal arterial tissues [12]. Among the various cytokines, HMGB1 has been studied widely in abdominal aortic artery aneurysms (AAAs). HMGB1 is specifically involved in endothelial cell inflammation and arterial stiffening [13] and is actively or passively released from injured or inflammatory cells [14,15]. Serum HMGB1 levels were significantly higher in patients with AAAs than in healthy controls [16]. Kohno et al. [15] reported that inhibiting HMGB1 suppressed AAAs. Also, serum HMGB-1 levels were significantly decreased following AAA treatment [16]. Thus, an investigation into the potential role of HMGB1 in IAs in regard to monitoring and treatment should be conducted by evaluating SNPs and HMGB1 expression levels [17].

Genes encode proteins through transcription and translation processes. Although significant differences in DNA sequences exist in patients with IAs, the levels of specific mRNAs or proteins may not differ. Thus, comprehensive studies on blood-based genetic variations and expression are necessary to delineate the effect of HMGB1 gene expression on IAs and discover its potential as an IA biomarker in actual clinical practice. Recently, we reported novel candidate SNPs associated with IAs, such as 1q31.2 (RGS18), 4q12, LINC01162-SP4, MYH13, and SLC47A1, in Korean patients diagnosed with IAs using a genome-wide association study (GWAS) implemented using the high-throughput imputation technique based on an Asian reference panel [18]. However, a more comprehensive investigation of SNP analyses and the expression of potential candidate genes has yet to be conducted. Here, we investigated the influence of HMGB1 polymorphisms on IAs based on previous GWAS datasets. We also investigated whether the expression of plasma HMGB1 mRNA and protein could be useful in predicting IA diagnosis before rupture.

## 2. Materials and Methods

### 2.1. HMGB1 Target Gene Association Analysis

This study was approved by the Institutional Review Board (Nos. 2017-9, 2018-6, and 2019-6) of the hospitals that participated in the GWAS. Informed consent was obtained from the patients or their relatives.

**Figure 1:**
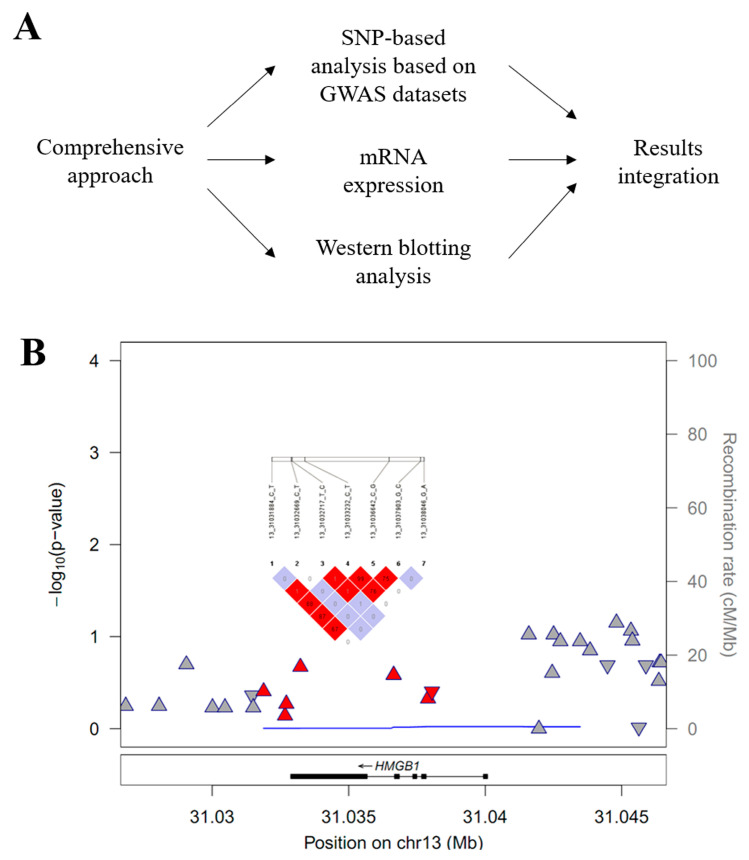
(**A**) Flow charts outlining the comprehensive analysis of HMGB1 polymorphisms associated with intracranial aneurysms (IAs) in this study. (**B**) A regional association plot of the HMGB1 region (13q12.3, chr13:31026642–31046642 of the rs3742305 SNP: chr13:31036642 bp ± 10 kb) associated with IAs. Triangles and reverse triangles indicate positive and negative effect sizes, respectively. Red colors indicate 500 base pair (bp) regions downstream and upstream of the HMGB1 gene, including seven single-nucleotide polymorphisms (SNPs). Gray colors indicate other SNPs. A pairwise linkage disequilibrium (LD, r^2^) indicates a structured block between each of the seven HMGB1 SNPs.

A flow chart of the study protocol is summarized in Figure 1A. GWAS datasets, including 250 patients with IAs and 296 controls, were used in the analysis [18]. The IA inclusion criteria were as follows: (1) adult patients over 18 years of age; (2) saccular aneurysm shape; (3) sporadic IA; and (4) patients without other cerebrovascular diseases, tumors, or neurodegenerative diseases. The control group consisted of cases that satisfied the following criteria: (1) patients whose absence of IAs was demonstrated by imaging tests; (2) no history of cerebrovascular disease, tumors, or neurodegenerative diseases; and (3) adults over 18 years of age [17].

Because our raw dataset was not from a randomized controlled study, the function “Batch Allele Consistency (BAC)” was requested to correct the genome-wide genotype data. BAC can be used to identify and remove SNPs with inconsistent genotypes due to shifts in intensity across samples that were processed in separate cases and control batches. Thus, our genotype data constructed by the Axiom™ Genotyping Array platform were corrected by a best-practices workflow involving eight steps using three main software programs, Axiom™ Analysis Suite v1.1.1, Applied Biosystems™ Array Power Tools (APT) v1.18, and the SNPolisher™ R package v3.0 (Thermo Fisher Scientific, Waltham, MA, USA). We then applied an Asian-specific reference panel (GRCh37/human genome 19 [hg19]) generated by the GenomeAsia 100K Project that was supported by the National Institutes of Health (NIH) National Heart, Lung and Blood Institute (NHLBI). Ten high-throughput variations were imputed in Michigan Imputation Server v1.5.7 (https://imputationserver.sph.umich.edu/index.html, accessed on 29 December 2022).

Raw genotype data were generated using the AxiomTM Asia Precision Medicine Research Array (PMRA) Kit (Thermo Fisher Scientific, Waltham, MA, USA) containing over 750,000 SNPs for East and South Asian populations based on the hg19 version (build 37). Before the imputation process, out of 802,688 SNPs, SNPs with mis-clustered genotypes, genotype call rates (GCRs) of <90%, minor allele frequencies (MAFs) of <0.01, and Hardy–Weinberg equilibrium (HWE) *p*-values of <1 × 10^−6^ at the pre-imputation stage were filtered out. Then, high-throughput imputation was applied using the Asian-based reference panel of the GenomeAsia 100K Project [19]; Minimac4 was used for SNP and genotype imputation, and Eagle v2.4 was used for pre-phasing works in the Michigan Imputation Server v1.5.7 (https://imputationserver.sph.umich.edu/index.html, accessed on 29 December 2022). Finally, our previous GWAS selected 6,105,212 SNPs out of a total of 7,333,746 sites for an imputation r^2^ score of ≥0.5, which passed the quality control tests requiring GCR ≥ 95%, MAF ≥ 0.01, and a HWE *p*-value ≥ 0.001. Detailed information regarding the genotype-imputation process is described elsewhere [17].

### 2.2. Real-Time Quantitative PCR and Western Blotting Analysis

Among the patients in the GWAS dataset, 24 were selected from the two groups to compare the expression of HMGB1 mRNA and protein while minimizing confounding factors that could affect the results, particularly clinical variables. Patients with previous inflammatory diseases, malignancies, collagen diseases, and chronic renal failure were not enrolled considering their potential effect on HMGB1 expression [15]. Real-time quantitative PCR (qPCR) was performed using Rotor-Gene Q analysis (Qiagen, Hilden, Germany). Genomic DNA was extracted from whole blood cells using the QIAamp DNA Blood Midi Kit (Qiagen, Hilden, Germany), and DNA concentrations were quantified in a 1 mm cuvette using an Eppendorf BioSpectrometer (Eppendorf, Hamburg, Germany). Each sample was analyzed using SYBR™ Green PCR master mix (Applied Biosystems, Foster City, CA, USA) according to the manufacturer’s protocol: 95 °C for 15 min, followed by 45 cycles of 30 s at 95 °C, 30 s at 60 °C, and 30 s at 72 °C. The PCR primer sequences were: HMGB1 forward ‘5-GCGAAGAAACTGGGAGAGATGTG-3′ and reverse ‘5-GCATCAGGCTTTCCTTTAGCTCG-3′; and GAPDH forward ‘5- GTCTCCTCTGACTTCAACAGCG-3′ and reverse ‘5-ACCACCCTGTTGCTGTAGCCAA -3′. Western blotting analysis was performed by lysing whole blood cells with RIPA buffer (Intronbio, Sungnam, Republic of Korea). Protein concentrations were measured using the BCA protein assay kit (Thermo Scientific, Fair Lawn, NJ, USA). Total protein (20 ug) was resolved by 10% sodium dodecyl protein-polyacrylamide gel electrophoresis, and the peptides were transferred to polyvinylidene difluoride membranes (Bio-Rad, Hercules, CA, USA) [20,21]. After blocking the membranes with a double blocker (T&I, Daejeon, Republic of Korea) for 1 h, they were incubated overnight at 4 °C with the primary antibodies anti-HMGB1 (Invitrogen, Carlsbad, CA, USA) and anti-albumin (Abcam, Cambridge, UK). After washing three times for 10 min in 0.1% Tween-20 in Tris-buffered saline (mM Tris-HCl [pH 7.6] and 30 mM NaCl), the membranes were incubated with horseradish peroxidase-conjugated secondary antibodies. The membrane blots were then exposed using Pierce™ ECL Western Blotting Substrate (Thermo Scientific, Fair Lawn, NJ, USA).

### 2.3. Statistical Analysis

Categorical variables are expressed as numbers with frequencies, and continuous variables as the means and standard deviations when describing demographic characteristics. Results involving qPCR and Western blotting analyses are presented as the medians and 25th–75th percentiles and were analyzed using the Mann-Whitney U test [9]. Statistical analysis was performed using SPSS V.21 (SPSS, Chicago, IL, USA) and MedCalc (www.Medcalc.org, accessed on 1 September 2024), with statistical significance indicated at a *p*-value < 0.05. Categorical variables are expressed as numbers and frequencies, and continuous variables are expressed as the means and standard deviations when describing demographic characteristics. qPCR and Western blotting results are presented as the medians and 25th–75th percentiles and were analyzed using the Mann–Whitney U test [22]. In addition, the genetic associations between targeted HMGB1 polymorphisms and IAs were adjusted for several confounding factors, including gender, hypertension, diabetes mellitus, hyperlipidemia, and principal component analysis values. Univariate and multivariate analyses were performed using SPSS V.21 (SPSS, Chicago, IL, USA) and MedCalc (www.Medcalc.org, accessed on 1 September 2024), with statistical significance indicated at *p* < 0.05.

## 3. Results

### 3.1. Baseline Characteristics of the Study Participants

The analysis of HMGB1 SNPs was conducted using previously updated GWAS datasets and included 546 subjects [18]. The mean age of the patients with IAs was older than that of the control group, and diabetes mellitus was observed more frequently in the control group than in the patients with IAs. Clinical characteristics and the plasma expression levels of mRNA and protein were not significantly different in the IA and control groups (Table 1).

### 3.2. High Mobility Group Box 1 (HMGB1) Target Gene Association Analysis

The detailed study processes, including the target gene association study, mRNA expression determination, and protein analysis by Western blotting, are described in Figure 1A. Regional associations within or around the 13q12.3 region are presented in Figure 1B. The HMGB1-targeted gene association study revealed seven SNPs, including rs1360485, rs185382445, rs2039338, rs1045411, rs3742305, rs2249825, and rs189034241, within or nearby the HMGB1 gene (Table 2). These SNPs are located on a 3-prime untranslated region (UTR-3) or intron region. Among these variants, two SNPs, rs1045411 (UTR-3) and rs3742305 (intron), showed a strong LD structure (r^2^ = 0.99). The rs1045411 SNP showed the highest association with IAs of the seven SNPs, but it was not statistically significant (odds ratio (OR) = 0.79, 95% confidence interval (CI): 0.54–1.15; *p* = 0.2138). Also, a difference of 0.035 was seen in MAF values between IA and control samples, probably due to the statistical underpower caused by the relatively small sample size. Comprehensively, none of the HMGB1 polymorphisms associated with IAs demonstrated a statistically significant relationship (adjusted *p* < 0.0016).

### 3.3. HMGB1 mRNA and Protein Analysis

**Figure 2:**
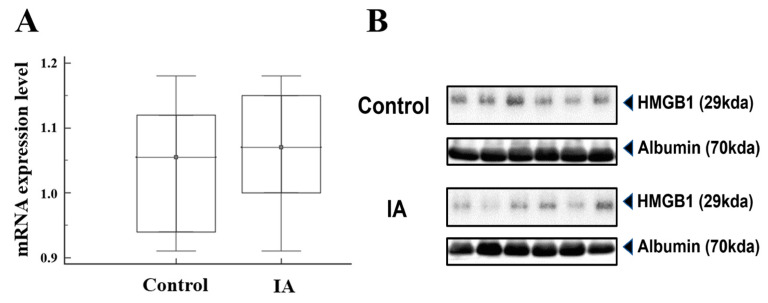
Comparative analysis of HMGB1 mRNA expression (**A**) and Western blots (**B**) between patients with intracranial aneurysms (IAs) and controls. Albumin was used as the loading control. The error bars indicate the median with the 25% and 75% percentiles.

Plasma mRNA and protein expression levels were quantified in 12 patients with IAs and 12 control subjects (Figure 2A). The median mRNA level (2^−ΔCt^) in patients with IAs was slightly higher than that in the control group [IA group, 1.07 (1.00–1.15) vs. control group, 1.05 (0.94–1.12)], but the difference was not statistically significant (*p* = 0.67). Regarding the Western blot analysis, patients with IAs did not differ significantly from the controls [IA group, 0.31 (0.13–0.57) vs. control group, 0.29 (0.13–0.60); *p* = 0.82] (Figure 2B and Supplemental Figure S1).

## 4. Discussion

Localized inflammatory infiltration of the IA wall due to hemodynamic stress triggers IA formation and growth [23]. Accordingly, the roles of inflammation in IA pathogenesis have been widely studied [23]. Sathyan et al. [23] investigated the associations of various pro-inflammatory and anti-inflammatory cytokine gene polymorphisms with IAs. Cytokines such as TNF-α (rs361525), interferon-gamma (IFN-γ), and IL-10 (rs1800871 and rs1800872) were significantly associated with IAs. Conversely, no such association was observed with IL-1 and IL-6 genes [23]. Bown et al. [24,25] reported that the A allele of IL-10 gene -1082G/A polymorphism (rs1800896) was linked to decreased secretion of IL-10 and an increased risk of AAA. In particular, the association between the AA genotype and AAA risk was established in an analysis of plasma from a large number of patients [26].

HMGB1 is a nonhistone protein expressed in eukaryotic cells [27,28]. HMGB1 plays a role in maintaining and sustaining inflammation by responding to early inflammatory mediators [29]. In the brain, HMGB1 induces inflammatory cells and astrocyte activation, further worsening inflammation by chemokine release [27,30]. Considering that an IA is formed due to inflammation and local tissue damage, HMGB1 may represent a potential diagnostic and therapeutic target for IAs. Cai et al. [31] reported that the genetic deletion or suppression of HMGB1 did not result in intimal hyperplasia, monocyte recruitment, or the proliferation of smooth muscle cells. Compared to UIAs, ruptured IAs exhibited a high level of HMGB1 monoclonal antibody staining within the IA wall [12]. Despite the potential of using HMGB1 for early IA diagnosis and determining the possibility of rupture, few studies have focused on HMGB1 SNPs or mRNA and protein expression as potential blood tests for IA diagnosis. Thus, we performed a comprehensive analysis of the associations of HMGB1 with IAs, including genetic variants, mRNA expression, and protein expression in the plasma, for the first time. HMGB1 genotype frequencies have rarely been reported to be associated with IAs, but studies have reported their associations with complications following SAH due to IA rupture [32,33]. Haruma et al. [34] reported cerebral vasoconstriction concomitant with decreased HMGB1 expression in the vascular smooth muscle cells of rats with SAHs. Treatment with an anti-HMGB1 monoclonal antibody attenuated cerebral vasoconstriction. These results suggest that HMGB-mediated inflammation plays an important role in delayed cerebral ischemia (DCI) caused by vasospasm. In a blood-based genetic study involving 147 patients with SAH, the minor G allele of rs2249825 was closely associated with the occurrence of DCI [27]. In this study, we identified seven HMGB1 SNPs, three of which (rs3742305, rs2249825, and rs189034241) were located in the intron. The remaining SNPs were located at or near the UTR. SNPs such as rs1045411, rs3742305, rs2249825, and rs1045411 increase the IA risk in patients with ischemic stroke [35]. Li et al. [35] reported that the rs2249825 variant was significantly associated with ischemic stroke. In particular, the infarct volume of patients with the G allele of rs2249825 was less than that of those with the CC genotype. However, unlike ischemic stroke, we did not detect meaningful HMGB1 variants with a possible link to IAs. Although IA has been increasingly recognized as a chronic inflammation-related disease and is closely associated with chronic inflammation [31,36], HMGB1 SNP genotyping could not be used to predict the occurrence of IAs.

Since circulating levels of inflammatory markers reflect disease presence and severity, we analyzed the differences in HMGB1mRNA and protein expression between patients with IAs and healthy control subjects. Sun et al. [37] reported that HMGB1 translocation was observed within 2 h after establishing an experimental SAH model with increased levels of mRNA and protein. Also, the administration of an anti-HMGB1 monoclonal antibody attenuated the enhanced vasocontractile properties of the cerebral arteries and activated microglia [34]. These findings indicate that extracellular HMGB1 contributes to the inflammatory responses after SAH, resulting in complications [37].

However, IA formation itself, not IA rupture, did not show a significant difference in HMGB1 expression levels in the plasma in our study. Three possibilities were considered for these results. First, the nature of the inflammation, such as systemic or chronic and local arterial inflammation in response to altered hemodynamic stress, could affect the outcome. IA formation is likely to be a local inflammatory process focused on the location of the cerebral artery and not systemic inflammation. Chyatte et al. [38] reported that an unruptured IA wall exhibited higher complement, immunoglobulin, macrophage, and T lymphocyte levels than control cerebral arteries, indicating extensive inflammatory and immunological responses limited to IA formation. Although HMGB1 is actively involved in chronic inflammation, such as that associated with adipose tissue in obesity [39], measuring HMGB1 expression in the blood is challenged by the difficulty of selecting a group of patients with a high probability of IA. Second, HMGB1 expression may vary depending on where the blood is collected. Chalouhi et al. [40] reported that the mean plasma concentrations of IL-8 and IL-17 were significantly increased in the IA lumen compared with levels in the femoral arteries of the same patients. In addition, monocyte chemoattractant protein was found to be highly expressed in unruptured aneurysms. These findings suggest that local inflammation may reflect inflammatory cell recruitment within the IA lumen. Thus, HMGB1 expression in blood obtained from the IA lumen may differ from that in other arteries, although no significant difference in HMGB1 expression was found in the systemic circulation, similar to our study results. Third, IAs are complex disorders influenced by multiple factors, such as genetic, biomechanical, and clinical factors. Altered hemodynamics have shown a close correlation with IA growth and rupture. Gholampour et al. [41] reported that IAs located at bifurcation sites showed an increased phase lag between the flow rate and pressure gradient graphs via computational fluid dynamic analysis, reflecting higher likelihoods of IA growth and rupture. In addition, when comparing the genome-based risk score system with the clinical factor-based risk factors, IA diagnoses did not increase, although the hazard ratio for the possibility of SAH increased from 0.63 (95% CI, 0.59–0.67) to 0.65 (95% CI, 0.62–0.69) [42]. Thus, future studies require more homogeneous clinical factors and should consider hemodynamic variability in explaining IA growth based solely on genetic risks.

The study had some limitations. First, we explored HMGB1 SNPs using previous IA GWAS datasets [17]. Although this was the first GWAS involving Korean patients with IAs, it was limited by the relatively small sample size. Second, we did not perform a comprehensive bioinformatics analysis of disease pathogenesis using data processing tools. Current efforts are targeted at improving the understanding of diseases using integrated approaches [43]. However, in the absence of significant differences in SNPs between IA and control samples, we could not proceed further. Third, we compared HMGB1 expression in blood samples, not IA tissues. Chronic inflammation reflected by HMGB1 expression may vary depending on the sample origin. Fourth, our results may be underpowered due to the relatively small number of enrolled patients, although we recruited both IA and control subjects using the random sampling process to reduce selection bias [13]. Also, our study had no other validation dataset, such as an independent study or cross-validation set. Thus, the association between HMGB1 expression and IAs should be analyzed in a large cohort of patients and tissues.

## 5. Conclusions

Our comprehensive analyses revealed no significant differences in HMGB1 SNPs, mRNA expression, or protein expression between patients with IAs and controls. However, the relatively limited data and the lack of external validation made it difficult to establish the diagnostic potential of HMGB1 in IAs using blood testing. For more definite scientific conclusions, additional studies should address this association in a large number of patients while considering more homogeneous clinical and hemodynamic backgrounds that potentially affect IA pathogenesis.

## Figures and Tables

**Table 1:** Clinical characteristics of the patients enrolled in the genome-wide association study (GWAS) datasets and a comparative analysis of plasma mRNA levels and Western blots of patients with intracranial aneurysms (IAs) and control subjects.

	GWAS Datasets			mRNA and Western Blotting		
Variables	IA (*n* = 250)	Control (*n* = 296)	*p*-Value	IA (*n* = 12)	Control (*n* = 12)	*p*-Value
Male	104 (41.6%)	142 (48.0%)	0.74	6 (50.0%)	5 (41.7%)	0.68
Age, years	59.3 ± 0.8	52.1 ± 1.0	<0.01	59.6 ± 11.6	56.3 ± 12.8	0.51
Hypertension	93 (37.2%)	88 (29.7%)	0.87	5 (41.7%)	4 (33.3%)	0.67
Diabetes mellitus	17 (6.8%)	37 (12.5%)	<0.01	2 (16.7%)	2 (16.7%)	1.00
Hyperlipidemia	29 (11.6%)	27 (9.1%)	0.48	3 (25.0%)	2 (16.7%)	0.62
Smoking	26 (10.4%)	37 (12.5%)	0.62	3 (25.0%)	2 (16.7%)	0.62

**Table 2:** Seven loci located nearby or on high mobility group box 1 (HMGB1) are associated with susceptibility to intracranial aneurysms.

Gene	Chr	SNP	Position	Function	M/m ^a^	MAF ^a^	HWEp ^a^	OR ^b^	L95 ^b^	U95 ^b^	*P * ^b^
HMGB1	13q12.3	rs1360485	31,031,884	Near UTR-3	T/C	0.144/0.171	0.838	0.86	0.60	1.22	0.394
HMGB1	13q12.3	rs185382445	31,032,669	Near UTR-3	C/T	0.018/0.017	1.000	0.84	0.32	2.18	0.7172
HMGB1	13q12.3	rs2039338	31,032,717	Near UTR-3	T/C	0.07/0.078	0.690	0.86	0.53	1.40	0.5355
HMGB1	13q12.3	rs1045411	31,033,232	UTR-3	C/T	0.124/0.159	0.384	0.79	0.54	1.15	0.2138
HMGB1	13q12.3	rs3742305	31,036,642	Intronic	C/G	0.124/0.157	0.386	0.81	0.56	1.17	0.2615
HMGB1	13q12.3	rs2249825	31,037,903	Intronic	G/C	0.102/0.122	0.593	0.86	0.57	1.29	0.4686
HMGB1	13q12.3	rs189034241	31,038,046	Intronic	G/A	0.024/0.025	1.000	1.44	0.62	3.34	0.3951

^a^ M/m indicates major and minor allele type, respectively. Minor allele frequencies are shown on the left for the case group and on the right for the control group. The Hardy–Weinberg equilibrium *p*-value was estimated in the control group. ^b^ Odds ratios (ORs), 95% confidence intervals (CIs: L95, lower 95% CI; U95, upper 95% CI), and *p*-values were estimated from the multivariate logistic regression model after adjustment for age, gender, hypertension, diabetes mellitus, hyperlipidemia, smoking status, and four genetic ancestry values.

## Data Availability

The data presented in this study are available from the corresponding author upon reasonable request. The data are not publicly available due to privacy and ethical restrictions.

## Supplementary Materials

The following supporting information can be downloaded at: https://www.mdpi.com/article/10.3390/brainsci14100969/s1, Figure S1: Original image of Figure 2B.
