# Supplementary figures and images for: Target Gene-Based Association Study of High Mobility Group Box Protein 1 in Intracranial Aneurysms in Koreans
Source: Brain Sci. 2024 Sep 26;14(10):969. doi: 10.3390/brainsci14100969 (PMC11505682; doi:10.3390/brainsci14100969)

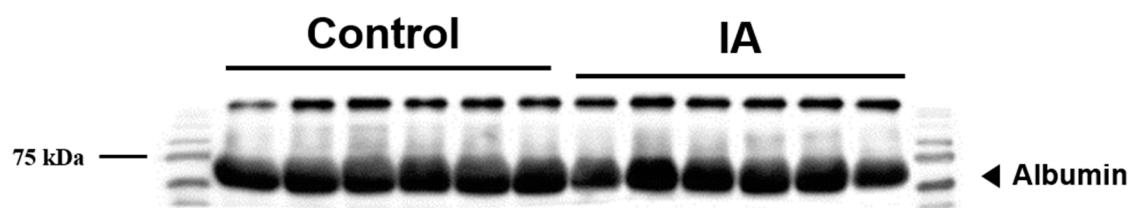

**Figure S1.** Original image of Figure 2B.

Supplement: Supplementary file 1 [file brainsci-14-00969-s001.zip › brainsci-3213174-supplementary.pdf]
